# The DEAD-Box Protein Dhh1 Promotes Decapping by Slowing Ribosome Movement

**DOI:** 10.1371/journal.pbio.1001342

**Published:** 2012-06-12

**Authors:** Thomas Sweet, Carrie Kovalak, Jeff Coller

**Affiliations:** Center for RNA Molecular Biology, Case Western Reserve University, Cleveland, Ohio, United States of America; University of Rochester School of Medicine and Dentistry, United States of America

## Abstract

Translational control and messenger RNA (mRNA) decay represent important control points in the regulation of gene expression. In yeast, the major pathway for mRNA decay is initiated by deadenylation followed by decapping and 5′–3′ exonucleolytic digestion of the mRNA. Proteins that activate decapping, such as the DEAD-box RNA helicase Dhh1, have been postulated to function by limiting translation initiation, thereby promoting a ribosome-free mRNA that is targeted for decapping. In contrast to this model, we show here that Dhh1 represses translation in vivo at a step subsequent to initiation. First, we establish that Dhh1 represses translation independent of initiation factors eIF4E and eIF3b. Second, we show association of Dhh1 on an mRNA leads to the accumulation of ribosomes on the transcript. Third, we demonstrate that endogenous Dhh1 accompanies slowly translocating polyribosomes. Lastly, Dhh1 activates decapping in response to impaired ribosome elongation. Together, these findings suggest that changes in ribosome transit rate represent a key event in the decapping and turnover of mRNA.

## Abstract 2

The highly conserved translational control protein Dhh1 promotes mRNA decapping by regulating a late step in translation in yeast.

## Author Summary

Translation of mRNA into protein and turnover of mRNA are two points at which cells can exert regulatory control of gene expression, thereby ensuring that the protein products are present in cells and tissues at the appropriate time and place. The DDX6 family of DEAD box helicases, exemplified by the yeast protein Dhh1, is a group of well-conserved eukaryotic proteins that regulate translation and mRNA decay. As DDX6 proteins are known to be important for diverse processes such as cellular stress responses, early embryonic development, and replication of some viruses, understanding their mechanism of action could be of broad significance to many fields. Previous studies suggest that Dhh1 and other DDX6-family proteins mainly regulate translation at the initiation stage, triggering sequestration and/or decapping of the mRNA. Our work expands the potential functions of Dhh1, showing that Dhh1 is also capable of inhibiting translation at later stages when ribosomes are already loaded onto mRNAs. This extended function for Dhh1 allows a more robust translational control, as inhibition at a late stage of translation can provide immediate stoppage of protein production, as well as affording the potential for storing mRNA already primed and loaded with ribosomes for subsequent rapid re-utilization.

## Introduction

Messenger RNA (mRNA) is targeted for destruction in a precise and regulated fashion. In eukaryotic cells, the digestion of the 3′ polyadenosine tail (deadenylation) is the first step, followed predominantly by removal of the mRNA cap and 5′→3′ exonucleolytic digestion or, rarely, 3′→5′ degradation catalyzed by the cytoplasmic exosome [1]. Decapping of mRNA, therefore, represents an important regulatory node in mRNA turnover and is, in most cases, both rate limiting and non-reversible [2]. In yeast, mRNA decapping is catalyzed by a single polypeptide encoded by *DCP2. DCP2* is conserved from yeast to humans, however it is becoming apparent that additional decapping activities exist in metazoans [3]. The rate at which an mRNA 5′ cap is removed is highly variable, and although not completely understood, the rate of Dcp2-dependent mRNA decapping is modulated by a suite of protein factors that facilitate the binding and catalytic activity of the decapping enzyme itself. Moreover, mRNA translation is critical in determining the overall level of decapping and stability of the mRNA [2]. mRNAs that initiate translation poorly are generally unstable and vice versa. The exact nature of the relationship between mRNA translation and decay is unclear, however it has been postulated that decapping activators may also function to promote mRNA turnover by monitoring mRNA translational status and/or promoting translation states that favor the decapping reaction. Of the many factors that influence mRNA decapping rates, the function of the DEAD-box RNA helicase Dhh1 most clearly ties mRNA decapping to protein synthesis.

Dhh1 was first shown to be involved in modulating mRNA decapping in yeast [4],[5]. At the same time, it was determined that Dhh1 homologues function as translational repressors in a variety of biological contexts. For example, the *Xenopus* ortholog of Dhh1, Xp54, was identified as a component of translationally silenced messenger ribonucleoprotein complexes (mRNPs) in *Xenopus* oocytes [6]. Moreover, the orthologous *Drosophila* protein, Me31b, is required for translational silencing of *oskar* mRNA and is, therefore, a critical determinant in defining the posterior pole in the fly embryo during development [7]. Subsequent studies indicated that Me31b also represents an important neurological factor through its regulation of *CaMKII* mRNA translation and association with the translational repressor, Fragile X Mental Retardation Protein (FMRP) [8],[9]. Furthermore, depletion of the human Dhh1 ortholog, RCK/p54 [10], or *Xenopus* Xp54 [11] leads to general derepression of mRNA translation. Finally, the role of yeast Dhh1 in promoting mRNA decapping was suggested to result from its role as a general translational repressor [12]. Together, these data demonstrate that Dhh1 and its homologues are a conserved family of translation regulatory proteins whose activity can lead to storage and/or destruction of translationally repressed mRNAs. Despite the widespread control on mRNA translation and turnover by Dhh1 proteins, the molecular mechanism by which it controls mRNA metabolism remains unclear.

Several pieces of evidence have supported a model that Dhh1 proteins alter the association of translation initiation complexes with mRNA, thereby rendering the cap accessible to the decapping machinery [12]. Consistent with this, a direct competition exists between the mRNA decapping and translation initiation machineries for the mRNA cap [13],[14]. Specifically, mRNA decapping rate is enhanced in vivo when translation initiation is impaired either in cis or trans. Moreover, the major cytoplasmic cap binding protein, eIF4E, competes with Dcp2 for association with the 5′ cap in vitro [15]. Thus, it has been proposed that association between translation initiation complexes and the mRNA must be antagonized before decapping can occur, a function that could be served by Dhh1. Two studies have provided evidence that *Xenopus* Xp54 complexes with the eIF4E inhibitor, eIF4E-T, thereby providing a possible model for how Dhh1 proteins could block eIF4E function [16],. In addition, experiments tethering Xp54 to an mRNA lead to the translational repression of capped mRNAs but not mRNAs lacking a 5′ cap or undergoing translation initiation using an internal ribosome entry site (IRES) element [18]. Lastly, recombinant Dhh1 inhibits 48S initiation complex formation in vitro [12].

The observation that decapping activators, including Dhh1, Pat1, and Lsm1, can be found in cytoplasmic aggregates called Processing bodies (P-bodies) has also provided support for a model in which translation initiation is blocked prior to mRNA decapping [19],[20]. P-bodies are proposed sites of mRNA decapping and degradation and encompass the full complement of decapping factors but are thought to be void of translation initiation factors and ribosomes [20]. In combination with the above work, this has led to a two-step model for mRNA decay in which deadenylation leads to the dissociation of mRNA from the translational apparatus and reorganization into a P-body where it is either stored or decapped and destroyed [20]. Importantly, the dissociation of ribosomes from the mRNA and mRNP remodeling have been hypothesized to be dependent on Dhh1 proteins [12]. Recent findings from a number of labs has, however, called into question the requirement for P-bodies in the translational repression and/or decay of mRNA, as these processes can be uncoupled from the accumulation of P-bodies in yeast and metazoans [21]–[23].

Under a common assumption that translation initiation is rate limiting for protein synthesis, repression of translation initiation prior to mRNA decapping would be predicted to result in ribosome run-off and decapping would occur predominately on ribosome-free mRNAs. In contrast, however, we have recently demonstrated that the majority of mRNA decapping occurs while mRNA maintains an association with polyribosomes, demonstrating that dissociation of mRNAs from ribosomes is not a prerequisite or general occurrence for mRNA decapping to occur [24],[25]. Based on this and additional evidence, we evaluated a role for Dhh1 in mediating a translational repression event that does not promote the loss of ribosome and mRNA association.

Here we show that Dhh1 functions in vivo primarily to repress mRNA translation and that its influence on decapping rate is predominantly a secondary effect. We demonstrate that Dhh1 inhibits mRNA translation in a manner independent of the translation initiation factors eIF4E and eIF3b. Consistent with the observation that mRNA decapping occurs on polyribosomes, tethering Dhh1 to an mRNA results in the accumulation of ribosomes on the mRNA. Moreover, endogenous Dhh1 protein associates with slowly moving polyribosomes. These data suggest that Dhh1 mediates a slowing of ribosome movement that may be a necessary first step before mRNA decapping can occur. Consistent with this, we show that slowing ribosome elongation in *cis* stimulates mRNA decapping in a Dhh1-dependent manner. Together, these data support a model that decapping of mRNA occurs on polyribosomes that have been impaired in ribosome transit in part by the activity of the general translational repressor Dhh1.

## Results

### Dhh1 Represses Translation Independent of mRNA Decapping

It has been extensively documented that Dhh1 and its orthologs are integral components of the decapping complex [5],[26]. Moreover, it has been observed that the homologs function as general repressors of mRNA translation [10]–[12]. The precise role for Dhh1 in this process has, however, remained elusive but has been suggested to involve remodeling of translation initiation factors at a step before 48S translation initiation complex formation on the mRNA [12],[16]–[18]. Due to the competition that exists for the mRNA 5′ cap between translation initiation factors and the decapping machinery, remodeling of the mRNP at the cap may be sufficient to explain the bipartite role Dhh1 appears to play in promoting both mRNA decapping and translational repression [12]. We wished to experimentally separate the two known functions of Dhh1 to evaluate the mechanism by which Dhh1 mediates translational repression and/or mRNA decay on an individual mRNA. Since little is understood about recruitment of Dhh1 to mRNA, we utilized a tethered-function approach to directly assay the functional consequences of Dhh1 binding to a reporter mRNA independent of its natural recruitment [27]. This assay has successfully been used to dissect the role of numerous RNA binding proteins in a variety of biological contexts [28]–[30].

**Figure 1:**
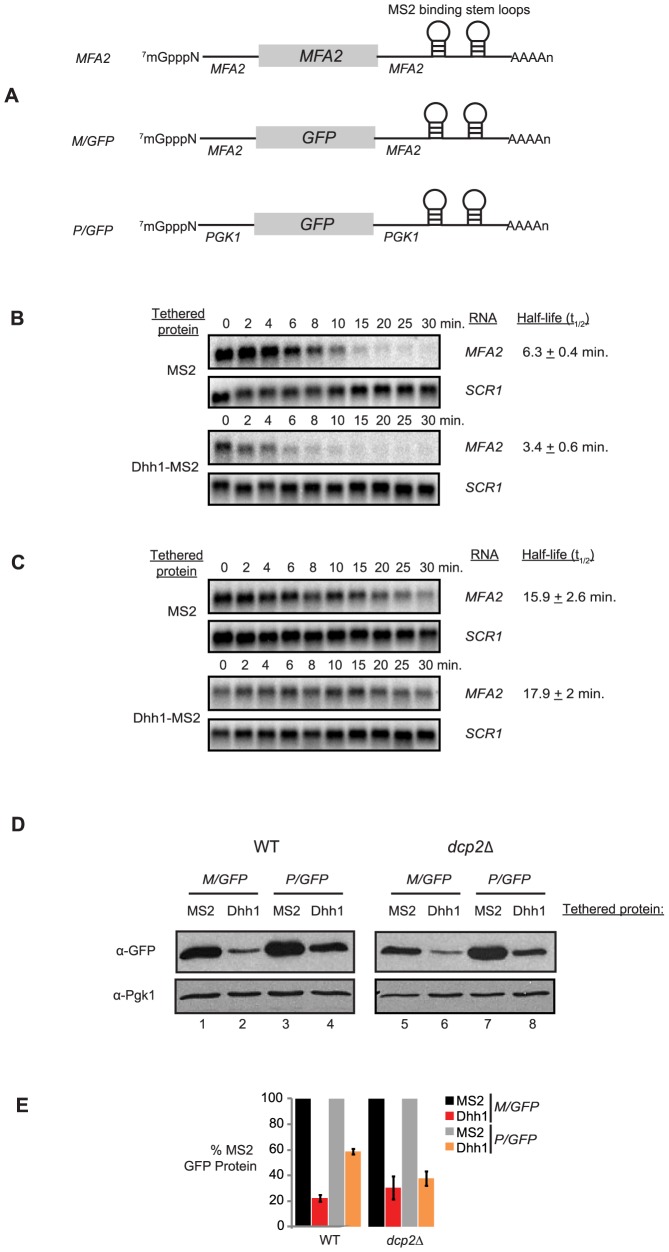
Tethered Dhh1 represses translation independent of decapping. (A) Diagram of reporter mRNAs Dhh1 was tethered to. Each reporter was expressed under control of the *GAL1* UAS, and each reporter has two MS2 binding stem-loops engineered in its 3′ UTR. First reporter, *MFA2*; second reporter, *M/GFP*; third reporter, *P/GFP*. Transcriptional shut-off analysis of *MFA2* in either wild-type cells (B) or *dcp2Δ* cells (C) expressing either MS2 alone or tethered Dhh1. RNA was isolated from cells collected at each time point and Northern blot for the reporter was performed. Blots were stripped and reprobed for *SCR1* as a loading control. Half-lives are reported in minutes to the right of the gels. (D) Western blot analysis of GFP from either *M/GFP* or *P/GFP* co-expressed with either MS2 alone or tethered Dhh1 in wild-type cells or *dcp2Δ* cells. Blots were stripped and reprobed for Pgk1 as a loading control. (E) Relative quantitation of GFP protein signal normalized to Pgk1 protein signal from Figure 1D. For a given experiment, signal with MS2 alone tethered was set to 100% and signal with Dhh1 tethered was expressed as a percentage of tethering MS2 alone.

The bacteriophage MS2 coat protein alone (MS2) or a protein chimera of Dhh1 and MS2 (Dhh1-MS2) were expressed from plasmid vectors along with reporter mRNA harboring MS2 RNA recognition elements in its 3′ UTR. Three different reporter mRNAs were used in various assays (Figure 1A). The first, *MFA2*, expresses the unstable *MFA2* mRNA with 3′ UTR MS2 binding sites [28]. The second and third represent *MFA2* and *PGK1* genes with their protein coding regions replaced by that of green fluorescence protein (*GFP*; Figure 1A; *M/GFP* and *P/GFP*, respectively). This combination of reporters allowed measurement of the consequence of tethering Dhh1 on both mRNA stability and translation (through protein output). We determined that Dhh1-MS2 was functionally active, as it was able to complement a strain deleted for endogenous *DHH1* (i.e. *dhh1Δ*) in assays for mRNA decapping (unpublished data).

We first evaluated whether Dhh1 altered mRNA decay when tethered to the 3′ UTR of a reporter mRNA. Wild-type (WT) cells expressing either MS2 or Dhh1-MS2 were evaluated for degradation of co-expressed *MFA2* reporter mRNA. Importantly, reporter mRNAs are expressed from the regulatable *GAL1* promoter, thereby permitting repression of reporter mRNA transcription and measurement of mRNA decay [13]. Cells were grown in the presence of galactose to induce reporter mRNA expression and, upon reaching mid-log phase, transcription was rapidly inhibited by replacing the media with glucose-containing media. Cells were harvested at indicated times and RNA isolated and analyzed by northern blot. As shown in Figure 1B, *MFA2* reporter mRNA is destabilized by Dhh1 tethered to its 3′ UTR. Specifically, the half-life of *MFA2* mRNA was reduced 2-fold by Dhh1-MS2 versus MS2 alone (3.4 min versus 6.3 min, respectively). Moreover, destabilization of the reporter mRNA required the MS2 binding sites, as *MFA2* mRNA lacking the sites decayed with a half-life of approximately 6 min, similar to endogenously expressed *MFA2* mRNA ([13]; unpublished data). These results establish that Dhh1, when associated with an mRNA through binding to its 3′ UTR, can accelerate the decay rate of the mRNA.

**Figure 2:**
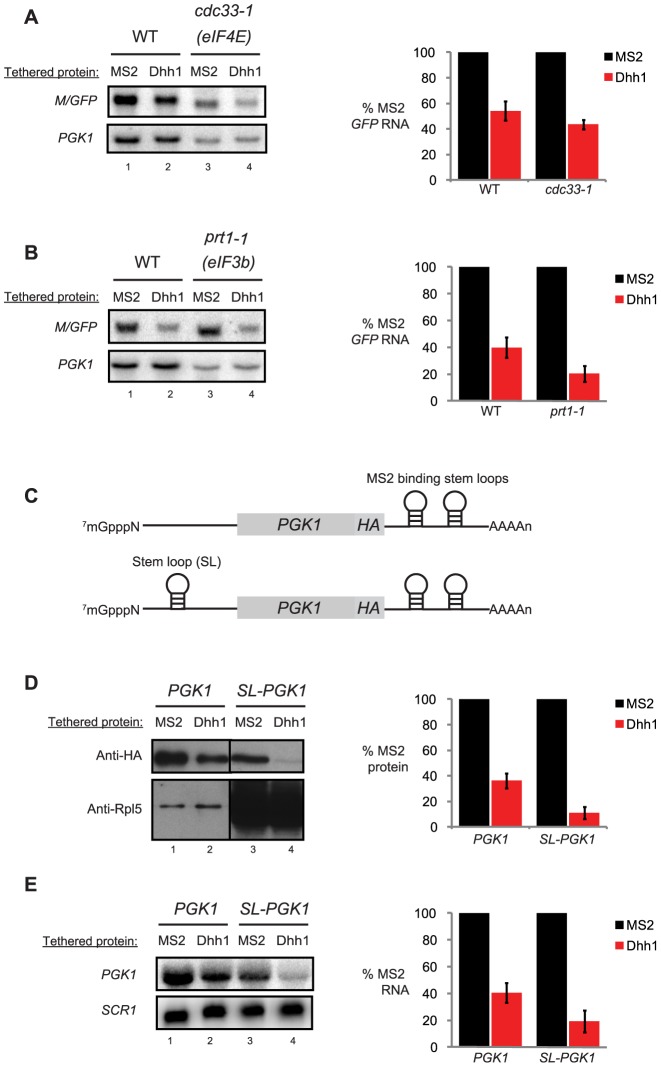
Tethered Dhh1 still functions under conditions in which translation initiation is limited. (A) Northern blot analysis of steady state *M/GFP* levels from both wild-type and *cdc33-1* (eIF4E mutant cells) cells co-expressing either MS2 alone or tethered Dhh1 grown at the restrictive temperature (37°C) for 1 h. Blots were first probed for the reporter, then were stripped and reprobed for endogenous *PGK1*. Relative quantitation of *M/GFP* signal is to the right of the gel. For a given experiment, signal with MS2 alone tethered was set to 100% and signal with Dhh1 tethered was expressed as a percentage of tethering MS2 alone. (B) Northern blot analysis of steady state *M/GFP* levels in both wild-type and *prt1-1* (eIF3b mutant cells) cells co-expressing either MS2 alone or tethered Dhh1 grown at the restrictive temperature (37°C) for 1 h. Blots were probed and quantitated as in Figure 2A. (C) Depiction of reporter mRNAs used in Figure 2D and 2E. Both reporters are derivatives of *PGK1pG* and as such are under control of the *GAL1* UAS; the pG tract has been replaced with two MS2 binding stem loops. Both reporters have also been engineered with an HA tag at the C-terminus of Pgk1 in order to distinguish the reporter from endogenous Pgk1 protein. The second reporter has a strong stem-loop engineered in the 5′ UTR. (D) Western blot analysis for Pgk1 and SL-Pgk1 proteins (with anti-HA) from wild-type cells co-expressing either MS2 alone or tethered Dhh1. Blots were stripped and reprobed with anti-Rpl5 antibody as a loading control. (E) Northern blot analysis for reporters in Figure 2C co-expressed with either MS2 alone or tethered Dhh1 in wild-type cells. Blots were stripped and reprobed for *SCR1* as a loading control.

*MFA2* mRNA is inherently unstable and its degradation is particularly sensitive to alterations in mRNA decapping [12]. We therefore evaluated whether the destabilization of *MFA2* reporter mRNA by tethered Dhh1 was mediated through changes in mRNA decapping rate. *MFA2* reporter mRNA decay was measured in the presence of either MS2 or Dhh1-MS2 in cells lacking mRNA decapping activity (i.e. *dcp2Δ*). *MFA2* reporter mRNA in the presence of MS2 coat protein alone was dramatically stabilized by the absence of Dcp2, similar to previous observations for endogenously expressed *MFA2* mRNA (Figure 1C; [2]). In contrast to our observation in wild-type cells, Dhh1-MS2 failed to lead to destabilization of *MFA2* reporter mRNA in the absence of *DCP2*, and the decay rate was essentially identical to that observed in cells expressing MS2 (Figure 1C). These results indicate that Dhh1 destabilizes mRNA through a step at or before mRNA decapping when associated by tethering.

We next set out to evaluate if Dhh1 can function as a translational repressor independent of its ability to promote mRNA decapping. To facilitate measurement of protein expression, *MFA2* and *PGK1* reporter mRNAs were generated in which their ORF was replaced with that of *GFP* (Figure 1A; *M/GFP* and *P/GFP*, respectively). Wild-type cells harboring either MS2 coat protein alone or Dhh1-MS2 and either *M/GFP* or *P/GFP* reporter genes were evaluated for GFP protein expression by Western blot analysis. As shown in Figure 1D, Dhh1-MS2 caused a 50%–80% reduction in protein expression when tethered to reporter mRNAs as compared to MS2 alone. Considering the observation that tethered Dhh1 also promotes mRNA decay (Figure 1B), one simple interpretation is that the reduced GFP protein level observed here is a consequence of reduced mRNA levels. To uncouple mRNA decay from a possible role for Dhh1 in repressing translation of the reporter mRNA, we repeated this analysis in the *dcp2Δ* strain, where tethering of Dhh1 did not alter mRNA decay rates (Figure 1C). In these cells, Dhh1-MS2 still mediated a dramatic decrease in GFP protein expression from both reporters (Figure 1D; GFP levels reduced 60%–70%). These data demonstrate that Dhh1 promotes repression of mRNA translation independent of promoting mRNA decapping when tethered to an mRNA, this is in agreement with recently published work [56].

Dhh1 has documented genetic and physical interactions with the deadenylase complex that, as the first step in mRNA degradation, removes the poly(A) tail from the mRNA [2],[5]. To establish whether tethering of Dhh1 modulates translational repression by simply recruiting the deadenylase to the mRNA and thereby facilitating poly(A) tail removal, we evaluated the effect of Dhh1-MS2 on *M/GFP* reporter mRNA translation in cells lacking *CCR4* (i.e. *ccr4Δ*), the gene expressing the catalytic subunit of the deadenylase complex [2]. We observed that similar to wild-type cells, tethering of Dhh1 facilitated translational repression of *M/GFP* mRNA in cells lacking *CCR4* (Figure S5), demonstrating that Dhh1 does not accelerate translational repression through removal of the poly(A) tail.

Finally, we established whether the function of Dhh1 in our assays requires a functional DEAD-box protein domain. Dhh1-MS2 in which key functional residues of the DEAD-box motif were mutated (DEAD to AAAD) was unable to reduce *M/GFP* reporter mRNA levels or GFP protein expression (Figure S1), in contrast to our observations for Dhh1-MS2 (Figures 1 and S1). These results demonstrate that Dhh1-MS2 requires the DEAD-box for function, similar to observations for endogenously expressed Dhh1 [31].

### Tethered Dhh1 Represses Translation Independent of eIF4E or the eIF3 Complex

Having established a robust assay to monitor the role of Dhh1 in repressing mRNA translation, we next set out to investigate the specific step of translation altered by Dhh1 function. Previous work from several labs suggested Dhh1 and its orthologs limit translation initiation prior to formation of the 48S pre-initiation complex [12], possibly by antagonizing eIF4E binding to the mRNA 5′ cap [16],[17],[32]. If Dhh1 indeed controls translation by blocking eIF4E function or 48S complex formation, loss of eIF4E or eIF3 function would be predicted to abrogate observed effects of tethered Dhh1 on *GFP* expression. Temperature-sensitive alleles of *CDC33* (*cdc33-1*, expressing eIF4E) or *PRT1* (*prt1-1*, expressing eIF3b) inactivate protein function and reduce mRNA translation to less that 5% of that observed in wild-type cells at the restrictive growth temperature [33],[34]. Importantly, residual mRNA translation allowed by these mutant alleles is required to be able to observe changes in mRNA translation of reporter mRNA. We were unable to use GFP protein levels to monitor changes in mRNA translation, however, since the 1-h incubation at the restrictive growth temperature sufficient to inactivate eIF4E or eIF3 function is short relative to the stability of GFP protein (∼7 h) [35]. Therefore, mRNA levels were used to reflect the translation status of the mRNA. This method to evaluate mRNA translation has been used previously [14] and is consistent with our observation that the function of Dhh1 on mRNA is primarily at the level of translation, and that mRNA decay represents a secondary consequence of translational control (Figure 1).

Isogenic wild-type or *cdc33-1* cells co-expressing the *M/GFP* reporter with either MS2 alone or Dhh1-MS2 were grown to log phase at the permissive temperature (24°C) and shifted to the restrictive temperature (37°C) for 1 h prior to harvesting cells and isolating RNA for Northern blot analysis. Growth of the mutant strain at the restrictive temperature resulted in a 4-fold reduction in steady state levels of both *M/GFP* reporter mRNA and endogenous *PGK1* mRNA in cells also expressing MS2 coat protein (Figure 2A, compare lanes 1 and 3). These data are consistent with previous observations [14] and demonstrate inactivation of eIF4E function under these growth conditions. Wild-type cells expressing Dhh1-MS2 displayed a 2-fold reduction in *M/GFP* mRNA levels compared to cells expressing MS2 alone (Figure 2A, compare lanes 1 and 2), consistent with the 2-fold reduction in decay rates by tethered Dhh1 (Figure 1). Relative to MS2 alone, Dhh1-MS2 resulted in an approximate 2-fold reduction in *M/GFP* reporter mRNA levels in *cdc33-1* cells expressing temperature-inactivated eIF4E (Figure 2A, compare lanes 3 and 4). These observations reveal that Dhh1 functions to robustly modulate reporter mRNA levels (through repressing mRNA translation) even in the absence of fully functional eIF4E and when translation initiation is severely abrogated, suggesting that Dhh1 does not function through modulating eIF4E activity.

The *Xenopus* homolog of Dhh1, Xp54, fails to repress translation of a reporter mRNA initiated from an internal ribosome entry site (IRES) [18]. Considering that IRES-mediated initiation does not require the eIF3 translation initiation complex, we hypothesized that it may be the target of Dhh1 function in repressing mRNA translation. To determine if eIF3 function is required for Dhh1-mediated effects on mRNA, we utilized cells harboring a temperature-sensitive allele of the gene expressing eIF3b (i.e. *prt1-1*). Importantly, this mutation in eIF3b leads to a significant disruption of the entire eIF3 complex and its function [36]. In *prt1-1* cells at the non-permissive temperature, endogenous *PGK1* mRNA levels are reduced approximately 4-fold (Figure 2B, lanes 1 and 3), demonstrating reduced eIF3b function as observed by others [14]. Interestingly, *M/GFP* reporter mRNA levels are insensitive to inactivation of eIF3b, suggesting that eIF3b is dispensable for the observed translation and mRNA turnover of this mRNA. Despite this, in eIF3b mutant cells Dhh1-MS2 was observed to still reduce *M/GFP* mRNA levels to approximately 20% relative to tethering MS2 alone (Figure 2B). This level of mRNA reduction is similar to that observed for Dhh1-MS2 in wild-type cells, indicating that Dhh1 function is unlikely through limiting the function of the eIF3 complex in promoting translation initiation.

Finally, we tested whether Dhh1 could modulate mRNA levels or translation of a reporter mRNA when translation of the mRNA is restricted in *cis*. mRNA translation was inhibited by the inclusion of a strong RNA secondary structure (i.e. stemloop; SL) in the 5′ UTR of a *PGK1* reporter that has been demonstrated to limit 48S ribosome scanning (Figure 2C; *SL-PGK1*) [12],[13]. The 5′ SL leads to reduced protein production from the *PGK1* reporter encoding a Pgk1-HA protein chimera (Figure 2D, compare lanes 1 and 3 where cells express MS2 alone). Indeed, when normalized to a loading control (i.e. ribosomal protein Rpl5), translation of *SL-PGK1* mRNA is less than 10% of the same reporter lacking the 5′ SL. In the presence of Dhh1-MS2, protein expression from both *PGK1* and *SL-PGK1* reporters was dramatically reduced relative to MS2 alone (Figure 2D; compare lanes 1 and 2 and lanes 3 and 4). Moreover, Dhh1-MS2 also led to a substantial decrease in steady state mRNA levels for both reporters (Figure 2E). These results demonstrate that despite an impairment in translation initiation at the level of ribosome scanning, Dhh1's function in inhibiting protein expression (and subsequently mRNA abundance) is not abrogated, and is as robust as that observed for reporter mRNAs undergoing translation in wild-type cells or in the absence of impediments presented by RNA structure. Together, these data indicate that repression of mRNA translation by Dhh1 is not mediated through modulation of eIF4E or eIF3 complex function, or 48S ribosome scanning.

### Dhh1 Causes Saturation of mRNA with Ribosomes

**Figure 3:**
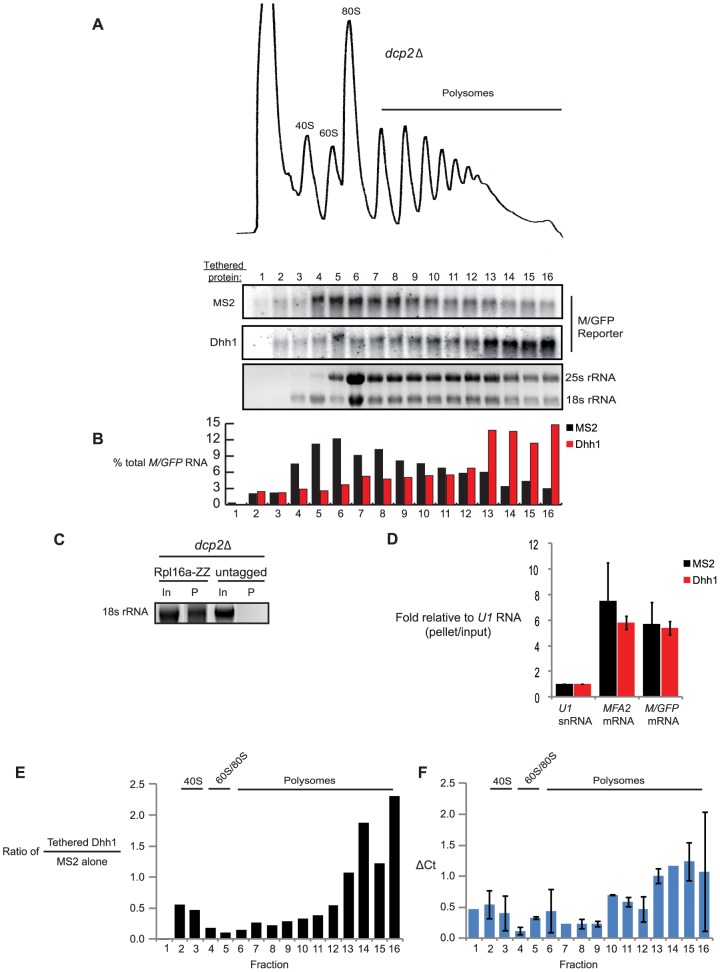
Tethering Dhh1 leads to accumulation of ribosomes on reporter mRNA. (A) Extracts from *dcp2Δ* cells expressing *M/GFP* and co-expressing either MS2 alone or tethered Dhh1 were separated by velocity sedimentation on sucrose gradients. RNA was extracted from each fraction and Northern blot was performed for *M/GFP.* The bottom panel is a representative ethidum bromide stained agarose gel showing the localization of 25S and 18S rRNA in sucrose gradients. (B) Quantification of signal from Figure 3A. Signal for each gradient was totaled and each fraction is represented as a percentage of the total. (C) Extracts from *dcp2Δ RPL16a-ZZ* cells co-expressing *M/GFP* and either MS2 alone or tethered Dhh1 were subjected to ribosome affinity purification followed by RNA isolation and agarose-formaldehyde gel electrophoresis. Ethidium bromide staining was used to visualize 18S rRNA (In, one-tenth input; P, pellet). (D) qRT-PCR for various RNAs from the ribosome affinity purification in Figure 3C to detect *U1*, *MFA2*, and *M/GFP*. ΔC_t_ between the pellet and the input were determined for each RNA, signal from *U1* in cells expressing MS2 alone was set to 1, and all other samples were expressed relative to *U1*. (E) Northern blot data from (A) were graphed as the ratio of *M/GFP* signal when Dhh1 was tethered to when MS2 was tethered for each fraction. (F) The same ribosome affinity purification was performed as in Figure 3C and 3D, except purified material was separated by velocity sedimentation on sucrose gradients. RNA was extracted from each fraction and *M/GFP* was detected by qRT-PCR. The ΔC_t_ was calculated for each fraction comparing the situation in which Dhh1 was tethered to the situation in which MS2 alone was tethered.

To further investigate the step of mRNA translation inhibited by Dhh1, the association of reporter mRNAs with ribosomes was monitored. Sucrose density centrifugation represents a powerful and unbiased biochemical technique used for decades to inspect perturbations in the various steps of translation. We evaluated *M/GFP* reporter mRNA in cells co-expressing either MS2 alone or Dhh1-MS2. Based on the loading of few ribosomes (Figure 3A and 3B; MS2), *M/GFP* reporter mRNA is ideally suited to observe changes in density based on alteration of its association with ribosomes. Mutant cells lacking mRNA decapping activity (i.e. *dcp2Δ*) were utilized to facilitate analysis of the effect of tethered Dhh1 on translation independent from secondary effects on mRNA turnover (Figure 1).

Cell extracts were layered on sucrose gradients and polyribosome complexes were separated by velocity sedimentation. During fractionation, absorbance at 254 nm was measured and “polyribosome traces” were generated (see Figure 3A). Total RNA was isolated from gradient fractions and *M/GFP* reporter mRNA was detected by northern blot. The polyribosome distribution of *M/GFP* mRNA from *dcp2Δ* cells expressing MS2 alone indicated that the mRNA associates predominantly with between 1 and 5 ribosomes (Figure 3A). In dramatic contrast, in the presence of Dhh1-MS2, the sedimentation of *M/GFP* mRNA shifted to a region deep within the gradient, consistent with heavy polyribosomes (Figure 3A). Importantly, Dhh1-MS2 did not lead to the accumulation of ribosome-free *M/GFP* mRNA detectable by sedimentation in non-ribosomal fractions 1 or 2, as would have been expected if tethered Dhh1 was inhibiting translation at initiation.

The detection of *M/GFP* mRNA in dense regions of the gradient when Dhh1 is tethered is consistent with but not conclusive evidence that ribosomes are abundantly associated with the mRNA. To directly determine the association of *M/GFP* mRNA with ribosomes, ribosomes were affinity purified from cell extracts and the associated RNA measured by qRT-PCR [37]. Yeast cells expressing a C-terminally tagged version of ribosomal protein Rpl16a (Rpl16a-ZZ) [37] were mutated to delete *DCP2* and then were used in subsequent experiments. Extracts were prepared from these cells expressing *M/GFP* reporter mRNA and either MS2 or Dhh1-MS2 and ribosomes immunoprecipitated using an anti-TAP antibody (see Materials and Methods). Visualization of co-purified RNA separated by agarose gel electrophoresis confirmed recovery of 18S rRNA from lysates containing tagged Rpl16a compared to an untagged control (Figure 3C). The association of specific mRNAs within the co-purified material was measured by qRT-PCR and normalized to the level of *U1* snRNA, a non-translated RNA that associates relatively inefficiently with ribosomes [37]. We observed that both endogenous and reporter mRNA can be efficiently co-purified relative to *U1* snRNA using this approach (Figure 3D). Moreover, reporter mRNA from cells expressing Dhh1-MS2 is co-purified to a similar extent as MS2 alone (Figure 3D; *M/GFP* mRNA; compare red and black bars). Importantly, co-purification of these mRNA targets is several hundred-fold enriched over that detected from similar experiments using lysates with untagged Rpl16a, indicating the specificity of the method (unpublished data). Our data suggest two important things. First, tethered Dhh1 does not lead to a large-scale dissociation of ribosomes from the mRNA, and second, the sedimentation of *M/GFP* reporter mRNA deep in polyribosome gradients (Figure 3A) must be due, in part, to its association with ribosomes. This latter observation is also inconsistent with the sedimentation of a large mRNP aggregate that lacks an association with ribosomes, such as P bodies [20].

To more rigorously establish that the dense sedimentation of *M/GFP* mRNA in the presence of Dhh1-MS2 represents ribosome-associated material, ribosomes were affinity purified from cell lysates as described above, and the co-purified material then subjected to sucrose density gradient sedimentation. Gradient fractions were collected and the abundance of reporter mRNA throughout the fractions measured by qRT-PCR. The ratio of mRNA present in gradient fractions from cells expressing Dhh1-MS2 versus MS2 was determined. *M/GFP* reporter mRNA showed a significant overrepresentation in dense polyribosome fractions in the presence of Dhh1-MS2 (Figure 3F; fractions 13–16) and a coordinate underrepresentation in the remainder of the fractions (Figure 3F; fractions 1–12). This observation is in strong correlation to that observed for this reporter mRNA subject directly to gradient sedimentation and analyzed by Northern blot (Figure 3A and quantified in 3E). The slightly reduced enrichment of reporter mRNA in dense gradient fractions in the presence of Dhh1-MS2 from cell lysates that were affinity purified reflects more efficient recovery of light polyribosomes over heavy polysomes by this approach (Figure S6; [37]). Notwithstanding, tethered Dhh1 causes the increased sedimentation of reporter mRNA in sucrose gradients and this material is clearly associated with ribosomes. Moreover, mRNA repressed in their translation by tethered Dhh1 appear to be associated with a larger number of ribosomes than during their basal metabolism and may indicate that Dhh1 functions to limit translation at some late step, perhaps at elongation, termination, or the poorly characterized ribosome recycling step.

### Endogenous Dhh1 Protein Associates with Slowly Moving Polyribosomes

Our data utilizing tethered-function analysis to analyze Dhh1 suggests that the tethered protein represses mRNA translation at a step after initiation and that it inhibits disassociation of ribosomes from mRNA. We predicted that if endogenously expressed Dhh1 were performing the same function, Dhh1 should be found associated with polyribosomes. We and others have documented, however, that Dhh1 sediments with the soluble RNP in sucrose gradients [12],[38]. We reasoned that the association of Dhh1 with polyribosomes in cells with active decay machinery and minimal cues for translational repression (i.e. mid-log phase cells undergoing exponential growth) may be transient and difficult to detect biochemically. To evaluate this hypothesis, cells were treated with formaldehyde in vivo to promote crosslinking and stabilize Dhh1-polysome complexes [39]. The sedimentation of Dhh1 with polysomes and other translation-associated mRNPs was then evaluated by sucrose gradient sedimentation.

**Figure 4:**
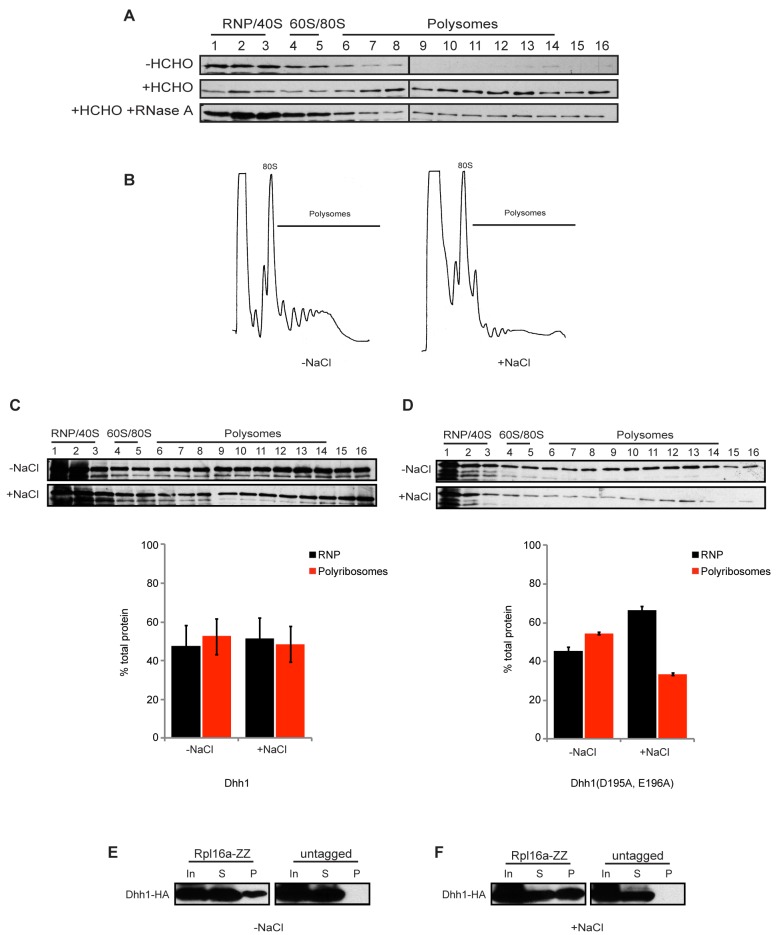
Dhh1 protein associates with slowly translocating polyribosomes. (A) Extracts from *dhh1Δ* cells expressing HBHT-tagged Dhh1 were separated by velocity sedimentation on 15%–45% sucrose gradients and protein was extracted from each fraction by TCA precipitation. SDS-PAGE was performed, protein was transferred to PVDF membrane, and Dhh1 was detected by Western blotting with anti-RGS-His antibody. −HCHO, without formaldehyde crosslinking; +HCHO, with formaldehyde crosslinking; +RNase A, with ribonuclease A. (B) Representative polyribosome traces from extracts of cells treated without (−NaCl) and with (+NaCl) 1 M NaCl. (C) Same analysis as in (A) for HBHT-Dhh1 association with polyribosomes from cells treated with or without 1 M NaCl. (D) Same analysis as in (C) of mutant Dhh1(D195A, E196A). (E) Ribosome affinity purification was performed on extracts from crosslinked cells resuspended in media without 1 M NaCl, expressing both *RPL16a-ZZ* and *DHH1-HA* or *DHH1-HA* alone (untagged). Shown is a Western blot probed for Dhh1 using anti-HA antibody. (In, one-tenth input; S, one-tenth supernatant; P, pellet). (F) same analysis as (E), but with cells treated with 1 M NaCl.

For this analysis, *dhh1Δ* cells expressing a plasmid-encoded, epitope-tagged Dhh1 protein (HBHT-Dhh1, [40]) were utilized. Importantly, HBHT-Dhh1 is fully functional and complements *dhh1Δ* cells for growth and the metabolism of *EDC1* mRNA (Figure S2). As shown in Figure 4A, in the absence of formaldehyde, HBHT-Dhh1 fails to co-sediment with polyribosomes, as previously observed [12],[38]. In contrast, after mild crosslinking, HBHT-Dhh1 is present in heavy sucrose gradient fractions, suggesting that it co-sediments with polyribosomes. Treatment of cell extracts with RNase A prior to centrifugation abrogates the co-sedimentation pattern, indicating that the association of Dhh1 with dense material on sucrose gradients is mediated by RNA contacts, consistent with its association with polyribosomes and its ability to bind RNA [41].

Our evidence indicates that tethered Dhh1 limits translation at a step after initiation and increases the sedimentation of reporter mRNA in sucrose gradients (Figure 3) and wild-type Dhh1 is associated with polyribosomes (Figure 4A). Based on these observations, we hypothesized that wild-type Dhh1 may also play a role in inhibiting ribosome elongation, termination, and/or ribosome recycling. In any case, it would be predicted that after a block in translation initiation, Dhh1-bound mRNA would retain a prolonged association with ribosomes. To measure the association of Dhh1 with polyribosomes after inhibition of translation, cells were treated with 1 M sodium chloride for 10 min prior to harvesting and polysome analysis. Exposure of cells to high salinity inhibits translation and results in ribosome run-off from mRNAs and loss of polyribosomes as measured by sucrose gradient centrifugation [42]. Even in the presence of low levels of formaldehyde, treatment of cells expressing HBHT-tagged Dhh1 led to a significant loss of polysomes, as anticipated (Figure 4B) [42]. Polysome analysis followed by Western blot demonstrated that HBHT-Dhh1 remained predominantly associated with dense sucrose gradient fractions after inhibition of translation by high salt (Figure 4C). In contrast, Dhh1 harboring a mutation in the DEAD-box that abrogates Dhh1 function in repressing translation (Figure S1) fails to remain associated with polyribosomes under salt stress (Figure 4D). Taken together, these data support that Dhh1 associates with polyribosomes and that it acts to restrict the dissociation of ribosomes from polyribosomes as measured by in vivo ribosome run-off analysis.

To confirm that the association of HBHT-Dhh1 with dense sucrose gradient fractions represents its association with polyribosomes, ribosomes were affinity purified from cells grown in the presence or absence of salt stress. Consistent with the co-sedimentation of Dhh1 with polyribosomes (Figure 4A), HBHT-Dhh1 co-purifies with ribosomes (Figure 4E). Moreover, after inhibition of translation with high salt, Dhh1 maintains an association with ribosomes (Figure 4F), consistent with its co-sedimentation with polysomes by sucrose gradient centrifugation.

### Rare Codons in Reporter mRNA Accelerates Decay in a Dhh1-Dependent Manner

**Figure 5:**
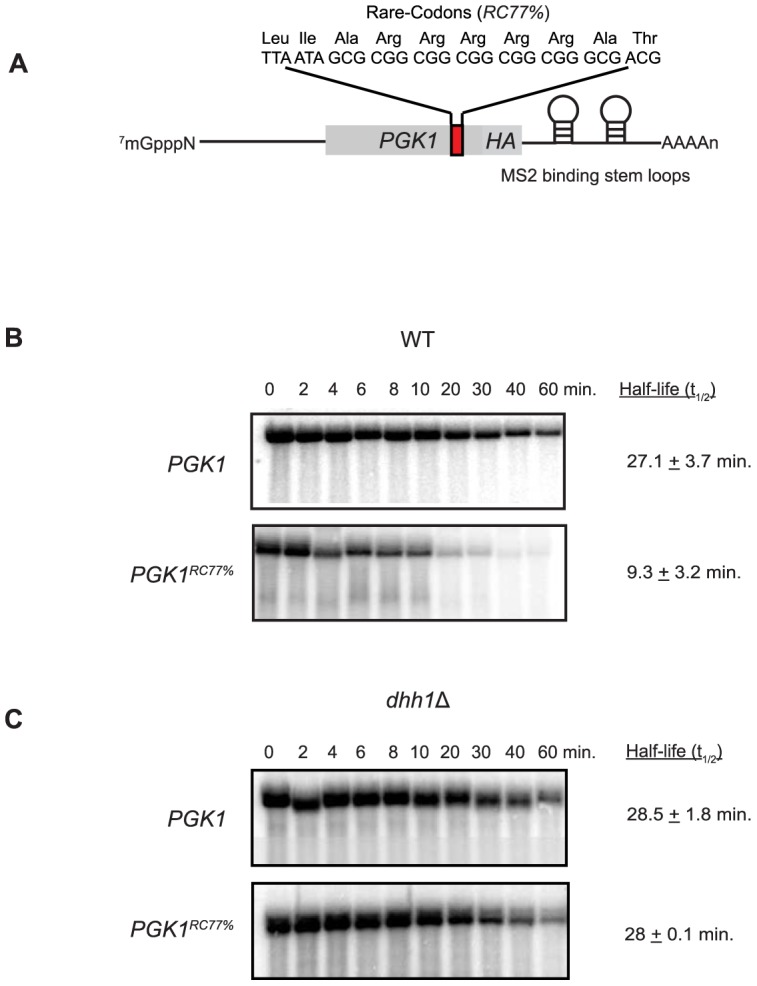
A stretch of rare codons engineered into *PGK1* accelerates mRNA decay in a Dhh1-dependent manner. (A) Rare codon-containing *PGK1* reporter (*PGK1^RC77%^*). The rare codon stretch utilized is depicted above the reporter. The percentage (77%) denotes the relative position of the start of the rare codon stretch in the ORF relative to the start codon. Transcriptional shut-off analysis was performed on *PGK1* and *PGK1^RC77%^* in wild-type (B) and *dhh1Δ* cells (C). RNA was isolated from each time point, and reporter level was assayed by Northern blot.

The observation that Dhh1 functions to limit ribosome run-off is consistent with Dhh1 inhibiting a step in translation subsequent to initiation and perhaps through limiting translation elongation. Moreover, as a consequence of Dhh1 function, mRNA decapping rate is enhanced leading to accelerated turnover of the mRNA (Figure 1). We hypothesized that inhibition of translation elongation by other means might also lead to a stimulation of mRNA decapping rate. To test this idea, a stretch of rare codons that restrict ribosome elongation [25] was inserted 77% into the coding region of a *PGK1* reporter gene (*PGK1^RC77%^*; Figure 5A). The rare codons greatly reduced Pgk1 protein expression to roughly 10% of wild-type *PGK1* reporter mRNA (Figure S3), demonstrating the inhibition of translation elongation. Importantly, *PGK1* reporter mRNA harboring the rare codons remains a substrate for mRNA decapping and 5′–3′ mRNA decay and is not targeted for No-go decay, as deletion of *DOM34* failed to significantly stabilize this reporter while deletion of factors important for 5′–3′ mRNA degradation significantly stabilized the mRNA [25]. Transcriptional shut-off analysis of both *PGK1* and *PGK1^RC77%^* in wild-type cells shows a significant destabilization of the mRNA dependent upon the rare codon stretch (Figure 5B). Specifically, the decay rate of *PGK1^RC77%^* mRNA is accelerated 3-fold versus *PGK1* mRNA lacking the rare codons (half-life of 9 min versus 27 min, respectively). These data demonstrate the inhibition of translation elongation can indeed elicit the acceleration of mRNA decapping.

If Dhh1 functions exclusively to inhibit translation elongation, the limitation of translation elongation mediated by the rare codons should bypass the need for Dhh1 in its rapid turnover of the reporter mRNA. We repeated the decay analysis for both *PGK1* and *PGK1^RC77%^* in cells in which *DHH1* was deleted (i.e. *dhh1*Δ). The decay of *PGK1* reporter mRNA was unaffected in *dhh1*Δ cells (Figure 5B and 5C), indicating that this mRNA is degraded in a Dhh1-independent manner. In contrast, the Lsm1–7 complex has a profound effect on *PGK1* mRNA stability (unpublished data). It is unclear why *PGK1* reporter mRNA is not a substrate for Dhh1 activity, but it will be an important mRNA in further elucidating Dhh1 function. Notwithstanding, *PGK1^RC77%^* mRNA was stabilized 3-fold in *dhh1*Δ cells compared to WT (Figure 5C), indicating that limiting ribosome movement on a reporter mRNA is not sufficient to bypass the requirement for Dhh1 function. Interestingly, the inhibition of ribosome elongation in *cis* does, instead, serve to render an otherwise Dhh1-insensitive mRNA into one that now responds to Dhh1 in the cell.

## Discussion

All mRNA succumbs to degradation; therefore, decay represents a default state in mRNA metabolism. The spectrum of mRNA half-lives observed for different mRNAs and in different cell types represents the acceleration or inhibition of the default rate of decay. One major factor that significantly contributes to the overall stability of an mRNA is its translatability [1],[43]. Indeed, an inverse correlation has been established wherein efficiently translated mRNAs display longer half-lives while poorly translated mRNAs are generally unstable. Competition for binding at or near the mRNA 5′ 7-methyl cap between the translation initiation factor eIF4E and the catalytic peptide of the decapping complex, Dcp2, is consistent with the observed inverse correlation between translation and mRNA decay. It is therefore generally assumed that modulating translational initiation is a key event in regulating the rate of mRNA decapping [14],[15].

The DEAD-box RNA helicase Dhh1 and its homologues have been implicated as active stimulators of mRNA decapping through dissociation of the translation initiation complex from mRNA. Specifically, Dhh1 proteins have been proposed to block initiation by interfering with eIF4E function [16],[32] or with eIF3-mediated 48S ribosomal complex assembly [12],[18]. Our previous work appeared to support these ideas [12]. Deletion of *DHH1* in combination with a second activator of mRNA decapping, *PAT1*, prevented broad repression of mRNA translation in response to glucose deprivation as analyzed by polysome analysis [12]. At that time, glucose deprivation was believed to cause widespread inhibition of translation initiation [44], and thus, our findings indicated that Dhh1 was required, in part, to modulate this process. Moreover, Dhh1 over-expression mediated a loss of bulk polysomes consistent with a general block to translation initiation. Finally, in vitro analysis of translation initiation complex assembly indicated that Dhh1 inhibited 48S complex formation on mRNA [12].

Advances in our understanding of mRNA metabolism call for new interpretations to previous observations. Recently, Arribere et al. showed that glucose deprivation leads to rapid and widespread degradation of most cellular mRNAs, rather than a general decrease in translation initiation [45]. The overall collapse in polyribosomes seen upon glucose deprivation is most likely a manifestation of this generalized decay phenomena. In our work from 2005 [12], the *RPL41a* mRNA was used to illustrate that mRNAs relocated from polyribosomes to non-polyribosome fractions upon glucose deprivation and that decay was not affected. Indeed, as a ribosomal protein gene, *RPL41a* belongs to the small class of mRNA not degraded following cell stress [45] but does dissociate from polyribosomes upon stress. Further work from our lab revealed that mRNAs targeted for decapping are not devoid of ribosomes, but rather, decapping occurs co-translationally while the mRNA is still associated with ribosomes [24],[25]. These observations highlight that a fundamental change in the association of an mRNA with ribosomes does not occur before mRNA decapping as previously hypothesized, but rather that mRNA decapping is co-translational.

Our findings presented here demonstrate that Dhh1 functions to repress mRNA translation, independent of any additional effect on promoting mRNA decapping (Figure 1). Moreover, Dhh1 functions at a step late in mRNA translation. Our data indicate that Dhh1 does not act through inhibiting eIF4E or eIF3 function (Figure 2A and 2B). Dramatically, when Dhh1 is tethered to a reporter, mRNA translation is repressed yet the mRNA co-sediments with denser polysomes that represent an increased association of the mRNA with ribosomes (Figure 3). Consistent with this, endogenous Dhh1 associates with polysomes, albeit in a transient manner. Finally, we show using saline-induced inhibition of translation initiation that Dhh1-polyribosome complexes dissociate from mRNA (i.e. run off) slowly (Figure 4). Together, these data demonstrate that Dhh1 is a bona fide translational repressor in vivo and that its function is consistent with a role in slowing ribosome movement on mRNA.

The function of Dhh1 in regulating translation post-initiation is consistent with phenomena observed in several additional biological contexts. First, two developmentally regulated mRNAs repressed on polyribosomes in *Drosophila* embryos, *oskar* and *nanos*, are inhibited for translation at some level by the Dhh1-homolog Me31b [7],[46]–[48]. Human *KRAS* mRNA is repressed on polyribosomes by let-7 miRNA in human cells [49], and this repression is partially attributed to RCK/p54 [10]. Interestingly, ribosome run-off of let-7-targeted *KRAS* mRNA occurs more slowly in response to a stress-induced translation initiation block [49], consistent with the repressed *KRAS* mRNP also being associated with slowly moving ribosomes. Finally, the documented purification of ribosomes with Dhh1 as well as its co-purification of translation elongation factor 1a in an RNA-independent manner [38] support Dhh1 as a repressor of a late step in mRNA translation. Interestingly, Fragile X Mental Retardation Protein (FMRP), a polysome-associated neuronal RNA binding protein with interactions with Me31b [9], was also recently found to regulate translation by inducing stalling of ribosomes on target mRNAs [50].

**Figure 6:**
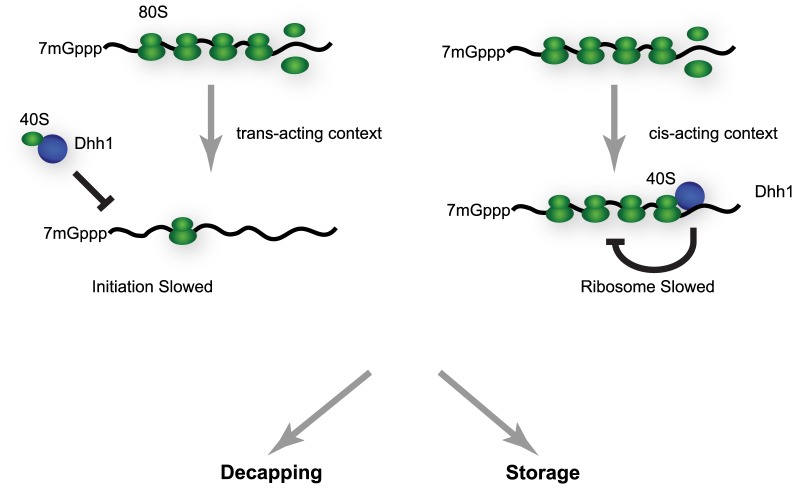
A novel function of Dhh1 is to repress a late step in translation. We hypothesize that Dhh1 may function directly on 40S ribosomal subunits based on our earlier findings in [12] and the documented interaction of Dhh1 with ribosomes (Figure 4 and [38]). If Dhh1 were to function on free 40S subunits, the consequence would be repression of translation at initiation, as was seen in [12] (depicted in the left side of the figure). Based on our findings in this article, action of Dhh1 on already assembled polyribosomes in vivo would lead to repression of translation at a late, post-initiation step (depicted in the right side of the figure). Repression of ribosome movement could either be direct repression of ribosomes or possibly further consolidation of already slowed ribosomes. Repressed polyribosomal mRNA can then either be decapped or stored depending on the biological context and activity of the decapping enzyme.

One potentially unifying theory of the data we have presented previously [12] and our current findings is that Dhh1 directly affects the function of the 40S ribosomal subunit. Indeed, we and others have observed that Dhh1 binds ribosomes [38]. Moreover, Dhh1 represses translation in vitro of an mRNA harboring the Cricket Paralysis Virus IRES, which requires only 40S ribosomes to initiate translation [12]. The context upon which Dhh1 binds to the 40S ribosomal subunit might affect which step in translation that appears to be inhibited (Figure 6). Interaction between Dhh1 and free 40S subunits could influence translation at early steps and manifest as an initiation block. This mechanism might be occurring both in vitro and during Dhh1 over-expression in cells [12]. In the context of an actively translating mRNA, however, Dhh1 interaction with 40S subunits might impede ribosome movement on mRNA as we have observed and implies a role for Dhh1 in inhibiting translation either during elongation, termination, or ribosome recycling. Additional experiments will be needed to define precisely how Dhh1 functions mechanistically, but the two sets of data need not be mutually exclusive.

Our data here suggest that Dhh1 may also function as a sensor for slowed translation elongation. Reducing ribosome elongation rate by the insertion of rare codons in a coding region of a reporter mRNA renders the mRNA unstable (Figure 5) and converts the mRNA into a substrate for Dhh1-mediated mRNA decay. This observation indicates that the accelerated decay in response to slowed ribosome elongation requires Dhh1. Interestingly, *dhh1Δ* cells also demonstrate an increased sensitivity to three general inhibitors of translation elongation (Figure S4), suggesting that in the absence of Dhh1, cells have a reduced ability to resolve the effects of a general inhibition of ribosome movement. The transient interaction of Dhh1 with polyribosomes (Figure 4) may reflect rapid sampling of polyribosome complexes by Dhh1, a common theme for biological sensors.

The role of Dhh1 as both a sensor of slowed ribosome movement and a mediator of translational repression is reminiscent of the function of another ATP-dependent RNA helicase, Upf1, in the decay of nonsense-containing mRNA. Upf1 is required for the recognition of aberrant translation termination events and in response to this event, mediates both translational repression and accelerated decapping of the mRNA [51]. For Dhh1-like proteins, one key regulatory event that may induce activity is removal of the mRNA 3′ poly(A) tail [17],[32]. Deadenlyation leads to the loss of poly(A) binding protein (Pab1) association with the mRNA and dramatic changes in the translational status of the mRNA are predicted to occur at many different levels, including elongation, termination and ribosome recycling [52]–[55]. In this light, we postulate that mRNA decapping serves an important role, preventing further translation from translationally impaired transcripts.

## Materials and Methods

### Yeast Strains and Growth Conditions

Yeast strains are listed in Table S1. Unless otherwise noted, all strains were grown at 24°C in synthetic media with the appropriate amino acids and either 2% galactose/1% sucrose, 4% glucose (for shutting off the *GAL1* UAS), or 2% glucose as appropriate. All cells were harvested at mid-log phase (OD_600_ = 0.4–0.55). Temperature-sensitive translation initiation mutant cells (yJC102, 104, 1011, or 1012) were shifted to the non-permissive temperature (37°C) for 1 h before harvesting. Cell stress experiments in Figure 4 were carried out by growing cells to mid-log phase, centrifuging the cells, and resuspending the cells in media with or without 1 M NaCl, then immediately adding formaldehyde as described below.

### Plasmids and Oligonucleotides

Details in Text S1 and Table S2.

### Transcriptional Shut-Off and Steady State RNA Northern Blot Analysis

Cells (yJC151, 327, or 330) expressing the appropriate plasmids were grown to mid-log phase in synthetic media containing 2% galactose/1% sucrose to allow expression of reporter mRNAs, then were centrifuged and resuspended in synthetic media without sugar. The 0 min time point was harvested, then glucose was added to a final concentration of 4% to shut off transcription. Cells were harvested at the time points indicated in each figure, then RNA was isolated by glass bead lysis followed by phenol/chloroform extraction and ethanol precipitation. 20–40 µg of total RNA from each time point were separated on 1.4% agarose-formaldehyde gels, transferred to nylon membranes, and probed overnight with ^32^P end-labeled oligonucleotides (listed in Table S2). RNAs were probed for using an oligonucleotide antisense to the MS2 binding sites (oJC1006), *PGK1* (oJC357), *EDC1* (oJC221), or *SCR1* (oRP100). Blots were exposed to PhosphorImager screens, scanned using a Storm 820 scanner, and quantified with ImageQuant software.

### Western Blot Analysis

Cells were grown to mid-log phase and harvested. Protein was isolated by resuspending cells in 200 µL 5 M urea, heating to 95°C for 2 min, vortexing cells with glass beads for lysis, adding 500 µL solution A (125 mM Tris-HCl pH 6.8, 2% SDS), vortexing 1 min, heating to 95°C for 2 min, and finally clearing extracts by centrifugation at 13,300 rpm for 2 min. Equivalent OD_280_ of extract was loaded onto 10% SDS polyacrylamide gels. Protein was transferred to PVDF membrane and blotted for various proteins (anti-HA, Covance; anti-Pab1, EnCor Biotechnology; anti-Rpl5; anti-Pgk1, Invitrogen; anti-RGS-His, Qiagen). Detection was carried out using Amersham ECL kit and exposing blots to Blue Ultra AutoRad film (ISC Bioexpress). Quantification was carried out by scanning the film and using ImageJ software.

### Polyribosome Analysis

Cells were harvested in 100 µg/mL cycloheximide. Cells used in Figure 4 were crosslinked at a final concentration of 0.25% formaldehyde for 5 min, then treated with 125 mM glycine for 5 min (Figures 4C through 4F) or 10 min (Figure 4A) to quench crosslinking. Cells were then lysed into 1× lysis buffer (10 mM Tris pH 7.4, 100 mM NaCl, 30 mM MgCl_2_, 0.5 mg/mL heparin, 1 mM DTT, 100 µg/mL cycloheximide) by vortexing with glass beads, and cleared using the hot needle puncture method followed by centrifugation at 2,000 rpm for 2 min at 4°C, then incubated in 1% Triton X-100 for 5 min on ice. In Figure 3A, 20 OD_260_ units were loaded on 15%–45% (w/w) sucrose gradients prepared on a Biocomp Gradient Master in 1× gradient buffer (50 mM Tris-acetate pH 7.0, 50 mM NH_4_Cl, 12 mM MgCl_2_, 1 mM DTT) and centrifuged at 41,000 rpm for 1 h and 13 min at 4°C in a Sw41Ti rotor. Gradients were fractionated using a Brandel Fractionation System and an Isco UA-6 ultraviolet detector. Fractions were precipitated overnight at −20°C using 2 volumes 95% ethanol. RNA/protein was pelleted at 14,000 rpm for 30 min, then pellets were resuspended in 500 µL LET (25 mM Tris pH 8.0, 100 mM LiCl, 20 mM EDTA) with 1% SDS. Fractions were then extracted once with phenol/LET, once with phenol/chloroform/LET, and then were precipitated with one-tenth volume of 7.5 M CH_3_COONH_4_ and 2 volumes 95% ethanol. RNA pellets were recovered by centrifugation at 14,000 rpm for 30 min. Pellets were washed once with 700 µL 75% ethanol, air dried, and resuspended in 1× sample buffer (200 mM MOPS pH = 7.0, 50 mM sodium acetate, 12.5 mM EDTA, 3.33% formaldehyde, 0.4 mg/mL ethidium bromide), and then samples were heated to 65°C for 10 min to denature RNA. The entire sample was then loaded on 1.4% agarose-formaldehyde gels and Northern analysis carried out as above. For Western blot analysis of protein from sucrose gradients, fractions were precipitated with a final concentration of 10% TCA, pellets were washed with 80% acetone, then allowed to air dry. Pellets were resuspended in 1× SDS-PAGE loading buffer, boiled, and loaded on 10% SDS polyacrylamide gels, then processed as in the section on Western blots.

### Ribosome Affinity Purification

*dcp2Δ* cells expressing a chromosomally ZZ-tagged version of Rpl16a (yJC1141) were grown to mid-log phase and harvested. Procedures were adapted from [37]. Cell lysis was performed as for polyribosome analysis by vortexing with glass beads in 1× lysis buffer without heparin. Samples were brought to 300 µL with 1× lysis buffer. Samples were then brought up to 592 µL with 2× binding buffer (100 mM Tris-HCl pH = 7.5, 24 mM Mg(CH_3_COO)_2_, 1 mM DTT, 100 µg/mL cycloheximide). Lysates were incubated at 4°C overnight with 4 µg anti-TAP antibody (Open Biosystems). The next morning, 1.5 mg protein-G Dynabeads (Invitrogen) were washed 3 times in a mixture of equal parts 1× lysis buffer and 2× binding buffer. The lysate from the night before was then incubated with protein-G Dynabeads for 1 h at 4°C. Pellets were washed 4 times in IXA-500 buffer (50 mM Tris-HCl pH = 7.5, 500 mM KCl, 12 mM Mg(CH_3_COO)_2_, 1 mM DTT, 100 µg/mL cycloheximide) and RNA/protein was eluted with elution buffer (50 mM Tris-HCl pH = 7.5, 0.5% SDS, 50 mM EDTA (pH = 8.0)) at 95°C for 5 min or TEV protease cleavage (100 U for 2 h in buffer C [20 mM Tris pH = 8.0, 140 mM KCl, 2 mM MgCl_2_, 5% glycerol, 0.5 mM DTT, 100 µg/mL cycloheximide]) for loading onto gradients (Figure 3F). RNA was isolated from one-tenth of the input or from the entire pelleted material by two phenol/chloroform extractions followed by chloroform extraction, then precipitated by sodium chloride and isopropanol. RNA was treated with 40 units of Roche DNase I, then extracted once with phenol/chloroform/LET and precipitated with sodium chloride and isopropanol, then resuspended in 15 µL of DEPC-treated dH_2_O.

### qRT-PCR

Reverse transcription was carried out using First Strand cDNA Synthesis Kit for Real-Time PCR from USB using random primers (or oJC1470 for 25S rRNA) and 1 µL of either a 32-fold dilution of input RNA from above or a 4-fold dilution of eluted RNA from each immunoprecipitation. qPCR was carried out using VeriQuest SYBR Green Master Mix (USB) in a StepOne Real Time PCR system (Applied Biosystems) and the following oligonucleotides: *GFP*, oJC1240, 1241; *MFA2*, oJC983, 984; *PGK1*, oJC985, 986; *U1*, oJC989, 990; 25S rRNA, oJC1470, 1471. Relative differences between samples were calculated using the ΔΔC_t_ method. A dilution series for each target ensured that we were within the linear range of the assay (unpublished data).

## Supporting Information

Figure S1 Mutation of the DEAD-box of Dhh1 to AAAD abrogates tethered Dhh1 function. (A) Wild-type cells co-expressing MS2 alone, tethered Dhh1, or mutated tethered Dhh1(D165A, E166A) with *M/GFP* reporter mRNA were grown to mid-log phase and protein was extracted from cells. Western blot for GFP was performed and quantification of GFP protein levels is provided in the histogram to the right of the gel. (B) From the same cells as in Figure S1A, RNA was extracted and analyzed by Northern blotting with radiolabeled oligonucleotides complementary to the MS2 binding sites as well as to a loading control RNA, *SCR1*. Relative quantification of *M/GFP* RNA signal is provided in the histogram to the right of the gel.
(EPS)

Figure S2 HBHT-tagged Dhh1 complements dhh1Δ cells for *EDC1* RNA levels. (A) Wild-type cells expressing an empty vector and *dhh1Δ* cells expressing either an empty vector, HBHT-Dhh1, or HBHT-Dhh1(D165A, E166A) were grown to mid-log phase, and then RNA was extracted from cells. *EDC1* mRNA levels were assessed by Northern blotting with a radiolabeled complementary oligonucleotide. Blots were then stripped and reprobed using a radiolabeled oligonucleotide complementary to *SCR1* RNA as a loading control. Quantification of *EDC1* RNA signal is provided in the histogram to the right of the gel. All samples were from the same gel/blot; the black bar between lanes 1 and 2 indicates that other lanes separated those two samples.
(EPS)

Figure S3 A rare codon stretch significantly reduces PGK1 translation. (A) Wild-type cells expressing either *PGK1* or *PGK1^RC77%^* were grown to mid-log phase, then protein was isolated from cells. Pgk1 protein levels were assayed by Western blot for the HA tag (*PGK1* reporter constructs were engineered to express a C-terminal HA tag for differentiation from endogenous Pgk1 protein). Blots were stripped and reprobed for poly(A) binding protein (PAB1) as a loading control. Note that 6.25 times more extract was loaded from cells expressing *PGK1^RC77%^* in order to see similar Pgk1 signal as from cells expressing *PGK1*. (B) Quantification of Pgk1 protein levels from the blot in Figure S3A.
(EPS)

Figure S4 *dhh1Δ* cells are sensitive to translation elongation inhibitors. (A) Wild-type or *dhh1Δ* cells were spread on synthetic complete media plates, then a piece of filter paper soaked in either H_2_O, cycloheximide, paromomycin, or hygromycin B was placed in the middle of the plate. Plates were incubated for several days and then pictures were taken to show relative sensitivities.
(TIF)

Figure S5 Tethered Dhh1 drives translational repression in the absence of the major yeast deadenylase. (A) Western blot analysis of GFP from extracts of *ccr4Δ* cells co-expressing *M/GFP* with either MS2 alone or tethered Dhh1. Blots were stripped and reprobed for Pgk1 as a loading control.
(EPS)

Figure S6 Ribosome affinity purification leads to underrepresentation of heavy polyribosomes. (A) Ribosome affinity purification followed by velocity sedimentation of purified material on sucrose gradients was performed exactly as in Figure 3F. RNA was extracted from each fraction and analyzed by qRT-PCR for 25S rRNA. Data are plotted as the ΔC_t_ between fraction 4 (80S subunits) and each fraction.
(EPS)

Table S1 Yeast strains.
(TIFF)

Table S2 Plasmids and oligonucleotides.
(TIFF)

Text S1 Supporting methods.
(DOC)

## Abbreviations

FMRP: Fragile X Mental Retardation Protein
*GFP*: green fluorescence protein
IRES: internal ribosome entry site
mRNA: messenger RNA
mRNP: messenger ribonucleoprotein complexes
P-bodies: Processing bodies
SL: stemloop
WT: wild-type

## References

[1] Franks T. M, Lykke-Andersen J (2008). The control of mRNA decapping and P-body formation.. Mol Cell.

[2] Coller J, Parker R (2004). Eukaryotic mRNA decapping.. Annu Rev Biochem.

[3] Li Y, Song M, Kiledjian M (2011). Differential utilization of decapping enzymes in mammalian mRNA decay pathways.. RNA.

[4] Fischer N, Weis K (2002). The DEAD box protein Dhh1 stimulates the decapping enzyme Dcp1.. EMBO J.

[5] Coller J. M, Tucker M, Sheth U, Valencia-Sanchez M. A, Parker R (2001). The DEAD box helicase, Dhh1p, functions in mRNA decapping and interacts with both the decapping and deadenylase complexes.. RNA.

[6] Ladomery M, Wade E, Sommerville J (1997). Xp54, the Xenopus homologue of human RNA helicase p54, is an integral component of stored mRNP particles in oocytes.. Nucleic Acids Res.

[7] Nakamura A, Amikura R, Hanyu K, Kobayashi S (2001). Me31B silences translation of oocyte-localizing RNAs through the formation of cytoplasmic RNP complex during Drosophila oogenesis.. Development.

[8] Hillebrand J, Pan K, Kokaram A, Barbee S, Parker R (2010). The Me31B DEAD-box helicase localizes to postsynaptic foci and regulates expression of a CaMKII reporter mRNA in dendrites of drosophila olfactory projection neurons.. Front Neural Circuits.

[9] Barbee S. A, Estes P. S, Cziko A. M, Hillebrand J, Luedeman R. A (2006). Staufen- and FMRP-containing neuronal RNPs are structurally and functionally related to somatic P bodies.. Neuron.

[10] Chu C. Y, Rana T. M (2006). Translation repression in human cells by MicroRNA-induced gene silencing requires RCK/p54.. PLoS Biol.

[11] Weston A, Sommerville J (2006). Xp54 and related (DDX6-like) RNA helicases: roles in messenger RNP assembly, translation regulation and RNA degradation.. Nucleic Acids Res.

[12] Coller J, Parker R (2005). General translational repression by activators of mRNA decapping.. Cell.

[13] Beelman C. A, Parker R (1994). Differential effects of translational inhibition in cis and in trans on the decay of the unstable yeast MFA2 mRNA.. J Biol Chem.

[14] Schwartz D. C, Parker R (1999). Mutations in translation initiation factors lead to increased rates of deadenylation and decapping of mRNAs in Saccharomyces cerevisiae.. Mol Cell Biol.

[15] Schwartz D. C, Parker R (2000). mRNA decapping in yeast requires dissociation of the cap binding protein, eukaryotic translation initiation factor 4E.. Mol Cell Biol.

[16] Minshall N, Reiter M. H, Weil D, Standart N (2007). CPEB interacts with an ovary-specific eIF4E and 4E-T in early Xenopus oocytes.. J Biol Chem.

[17] Minshall N, Thom G, Standart N (2001). A conserved role of a DEAD box helicase in mRNA masking.. RNA.

[18] Cooke A, Prigge A, Wickens M (2010). Translational repression by deadenylases.. J Biol Chem.

[19] Eulalio A, Behm-Ansmant I, Izaurralde E (2007). P bodies: at the crossroads of post-transcriptional pathways.. Nat Rev Mol Cell Biol.

[20] Parker R, Sheth U (2007). P bodies and the control of mRNA translation and degradation.. Mol Cell.

[21] Decker C. J, Teixeira D, Parker R (2007). Edc3p and a glutamine/asparagine-rich domain of Lsm4p function in processing body assembly in Saccharomyces cerevisiae.. J Cell Biol.

[22] Eulalio A, Behm-Ansmant I, Schweizer D, Izaurralde E (2007). P-body formation is a consequence, not the cause, of RNA-mediated gene silencing.. Mol Cell Biol.

[23] Sweet T. J, Boyer B, Hu W, Baker K. E, Coller J (2007). Microtubule disruption stimulates P-body formation.. RNA.

[24] Hu W, Petzold C, Coller J, Baker K. E (2010). Nonsense-mediated mRNA decapping occurs on polyribosomes in Saccharomyces cerevisiae.. Nat Struct Mol Biol.

[25] Hu W, Sweet T. J, Chamnongpol S, Baker K. E, Coller J (2009). Co-translational mRNA decay in Saccharomyces cerevisiae.. Nature.

[26] Simon E, Camier S, Seraphin B (2006). New insights into the control of mRNA decapping.. Trends Biochem Sci.

[27] Coller J, Wickens M (2007). Tethered function assays: an adaptable approach to study RNA regulatory proteins.. Methods Enzymol.

[28] Coller J. M, Gray N. K, Wickens M. P (1998). mRNA stabilization by poly(A) binding protein is independent of poly(A) and requires translation.. Genes Dev.

[29] Lykke-Andersen J, Shu M. D, Steitz J. A (2000). Human Upf proteins target an mRNA for nonsense-mediated decay when bound downstream of a termination codon.. Cell.

[30] Pillai R. S, Artus C. G, Filipowicz W (2004). Tethering of human Ago proteins to mRNA mimics the miRNA-mediated repression of protein synthesis.. RNA.

[31] Cheng Z, Coller J, Parker R, Song H (2005). Crystal structure and functional analysis of DEAD-box protein Dhh1p.. RNA.

[32] Minshall N, Standart N (2004). The active form of Xp54 RNA helicase in translational repression is an RNA-mediated oligomer.. Nucleic Acids Res.

[33] Welch E. M, Jacobson A (1999). An internal open reading frame triggers nonsense-mediated decay of the yeast SPT10 mRNA.. EMBO J.

[34] Altmann M, Trachsel H (1989). Altered mRNA cap recognition activity of initiation factor 4E in the yeast cell cycle division mutant cdc33.. Nucleic Acids Res.

[35] Mateus C, Avery S. V (2000). Destabilized green fluorescent protein for monitoring dynamic changes in yeast gene expression with flow cytometry.. Yeast.

[36] Phan L, Schoenfeld L. W, Valasek L, Nielsen K. H, Hinnebusch A. G (2001). A subcomplex of three eIF3 subunits binds eIF1 and eIF5 and stimulates ribosome binding of mRNA and tRNA(i)Met.. EMBO J.

[37] Halbeisen R. E, Scherrer T, Gerber A. P (2009). Affinity purification of ribosomes to access the translatome.. Methods.

[38] Drummond S. P, Hildyard J, Firczuk H, Reamtong O, Li N (2011). Diauxic shift-dependent relocalization of decapping activators Dhh1 and Pat1 to polysomal complexes.. Nucleic Acids Res.

[39] Valasek L, Szamecz B, Hinnebusch A. G, Nielsen K. H (2007). In vivo stabilization of preinitiation complexes by formaldehyde cross-linking.. Methods Enzymol.

[40] Tagwerker C, Flick K, Cui M, Guerrero C, Dou Y (2006). A tandem affinity tag for two-step purification under fully denaturing conditions: application in ubiquitin profiling and protein complex identification combined with in vivo cross-linking.. Mol Cell Proteomics.

[41] Dutta A, Zheng S, Jain D, Cameron C. E, Reese J. C (2011). Intermolecular interactions within the abundant DEAD-box protein Dhh1 regulate its activity in vivo.. J Biol Chem.

[42] Melamed D, Pnueli L, Arava Y (2008). Yeast translational response to high salinity: global analysis reveals regulation at multiple levels.. RNA.

[43] Jacobson A, Peltz S. W (1996). Interrelationships of the pathways of mRNA decay and translation in eukaryotic cells.. Annu Rev Biochem.

[44] Ashe M. P, De Long S. K, Sachs A. B (2000). Glucose depletion rapidly inhibits translation initiation in yeast.. Mol Biol Cell.

[45] Arribere J. A, Doudna J. A, Gilbert W. V (2011). Reconsidering movement of eukaryotic mRNAs between polysomes and P bodies.. Mol Cell.

[46] Cougot N, van Dijk E, Babajko S, Seraphin B (2004). ‘Cap-tabolism’.. Trends Biochem Sci.

[47] Braat A. K, Yan N, Arn E, Harrison D, Macdonald P. M (2004). Localization-dependent oskar protein accumulation: control after the initiation of translation.. Dev Cell.

[48] Clark I. E, Wyckoff D, Gavis E. R (2000). Synthesis of the posterior determinant Nanos is spatially restricted by a novel cotranslational regulatory mechanism.. Curr Biol.

[49] Maroney P. A, Yu Y, Fisher J, Nilsen T. W (2006). Evidence that microRNAs are associated with translating messenger RNAs in human cells.. Nat Struct Mol Biol.

[50] Darnell J. C, Van Driesche S. J, Zhang C, Hung K. Y, Mele A (2011). FMRP stalls ribosomal translocation on mRNAs linked to synaptic function and autism.. Cell.

[51] Isken O, Kim Y. K, Hosoda N, Mayeur G. L, Hershey J. W (2008). Upf1 phosphorylation triggers translational repression during nonsense-mediated mRNA decay.. Cell.

[52] Searfoss A, Dever T, Wickner R (2001). Linking the 3′ poly(A) tail to the subunit joining step of translation initiation: relations of Pab1p, eukaryotic translation initiation factor 5B (Fun12p), and Ski2p-Slh1p.. Mol Cell Biol.

[53] Bonderoff J. M, Lloyd R. E (2010). Time-dependent increase in ribosome processivity.. Nucleic Acids Res.

[54] Ivanov P. V, Gehring N. H, Kunz J. B, Hentze M. W, Kulozik A. E (2008). Interactions between UPF1, eRFs, PABP and the exon junction complex suggest an integrated model for mammalian NMD pathways.. EMBO J.

[55] Cosson B, Couturier A, Chabelskaya S, Kiktev D, Inge-Vechtomov S (2002). Poly(A)-binding protein acts in translation termination via eukaryotic release factor 3 interaction and does not influence [PSI(+)] propagation.. Mol Cell Biol.

[56] Carroll JS, Munchel SE, Weis K (2011). The DEAD/H box ATPase Dhh1 functions in translational repression, mRNA decay, and processing body dynamics.. J Cell Biol.

