# Supplementary figures and images for: The DEAD-Box Protein Dhh1 Promotes Decapping by Slowing Ribosome Movement
Source: PLoS Biol. 2012 Jun 12;10(6):e1001342. doi: 10.1371/journal.pbio.1001342 (PMC3373615; doi:10.1371/journal.pbio.1001342)

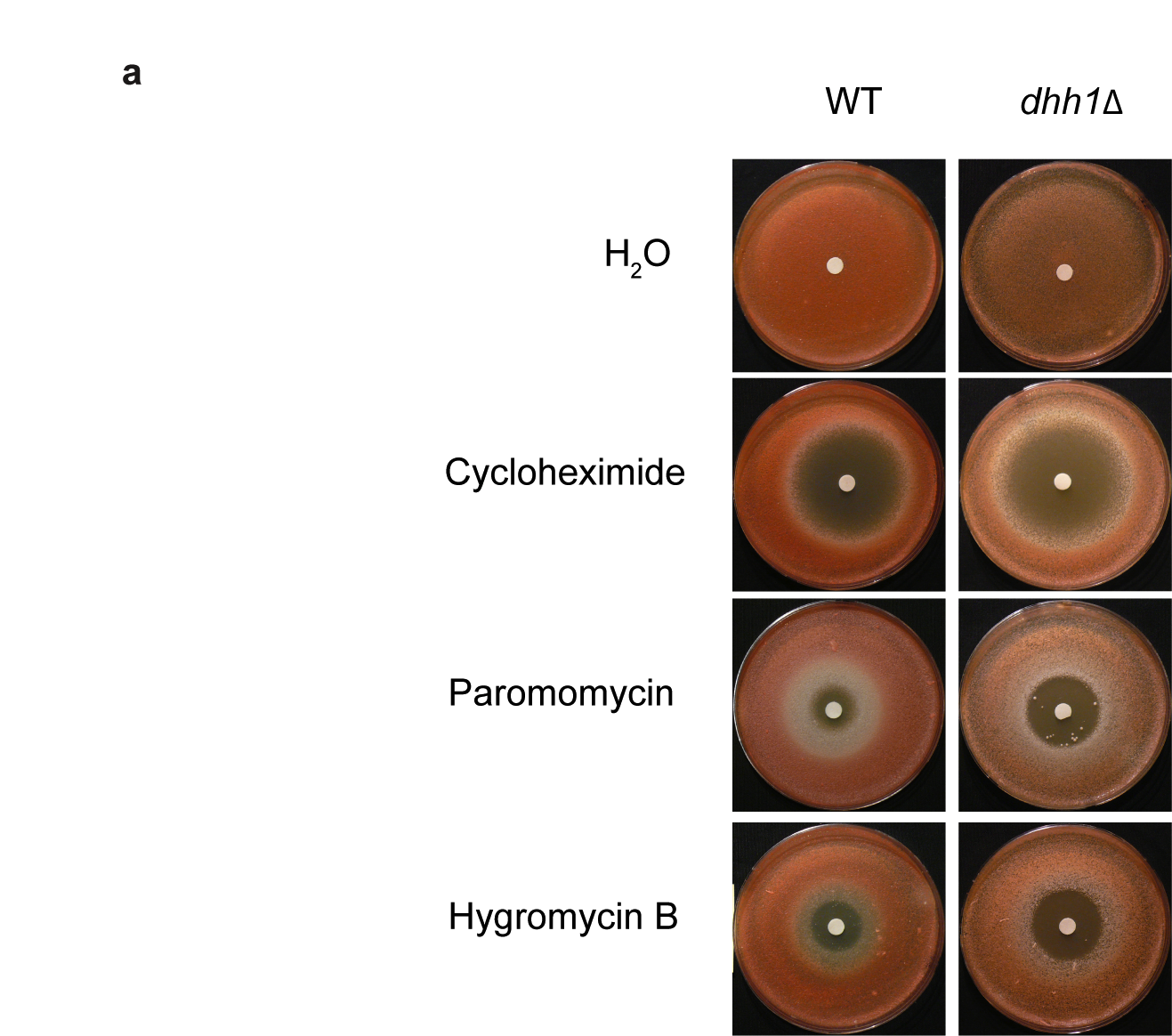

Supplement: Figure S4 — dhh1Δ cells are sensitive to translation elongation inhibitors. (A) Wild-type or dhh1Δ cells were spread on synthetic complete media plates, then a piece of filter paper soaked in either H2O, cycloheximide, paromomycin, or hygromycin B was placed in the middle of the plate. Plates were incubated for several days and then pictures were taken to show relative sensitivities. (TIF) [file pbio.1001342.s004.tif]

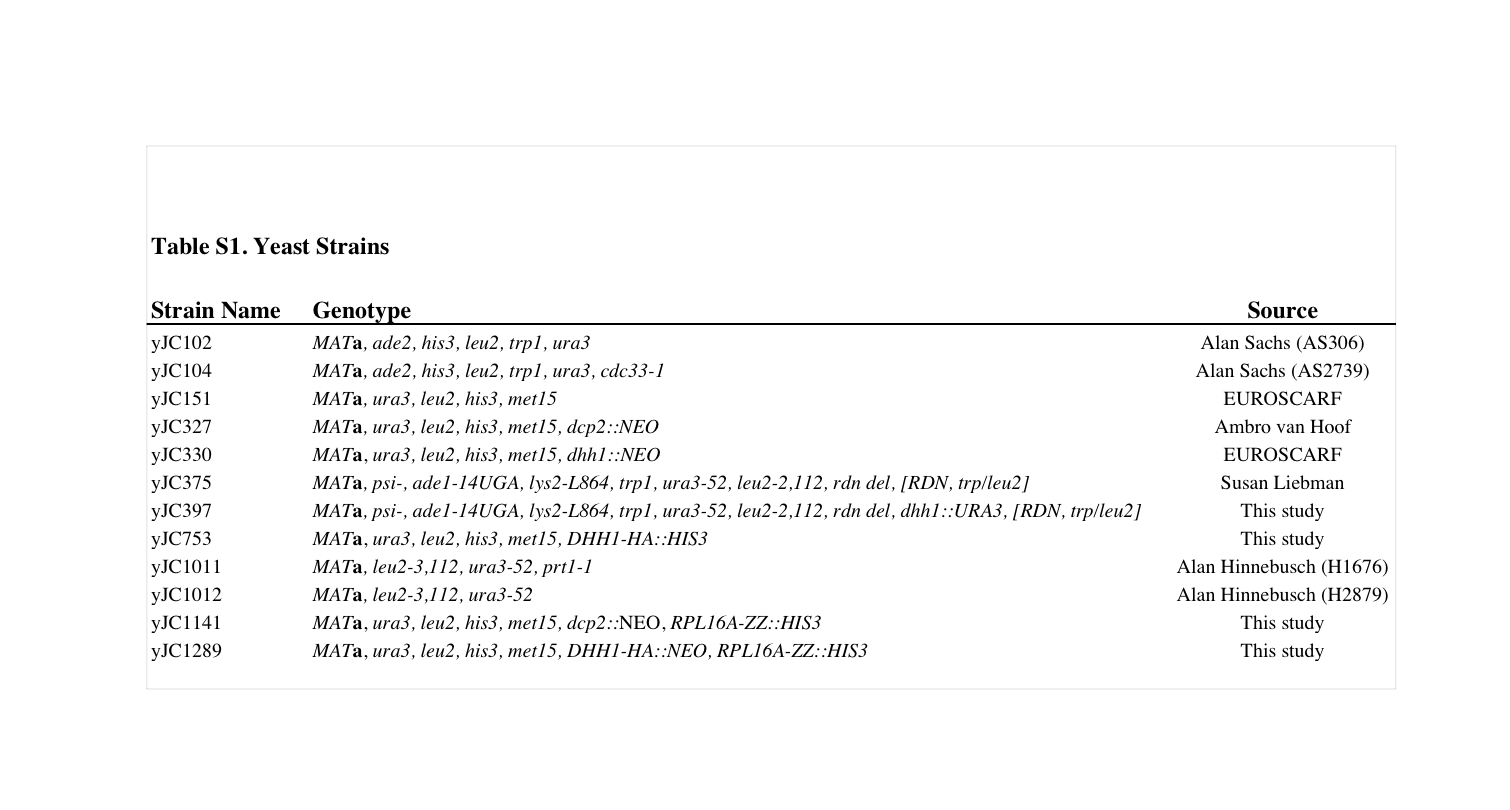

Supplement: Table S1 — Yeast strains. (TIFF) [file pbio.1001342.s007.tiff]

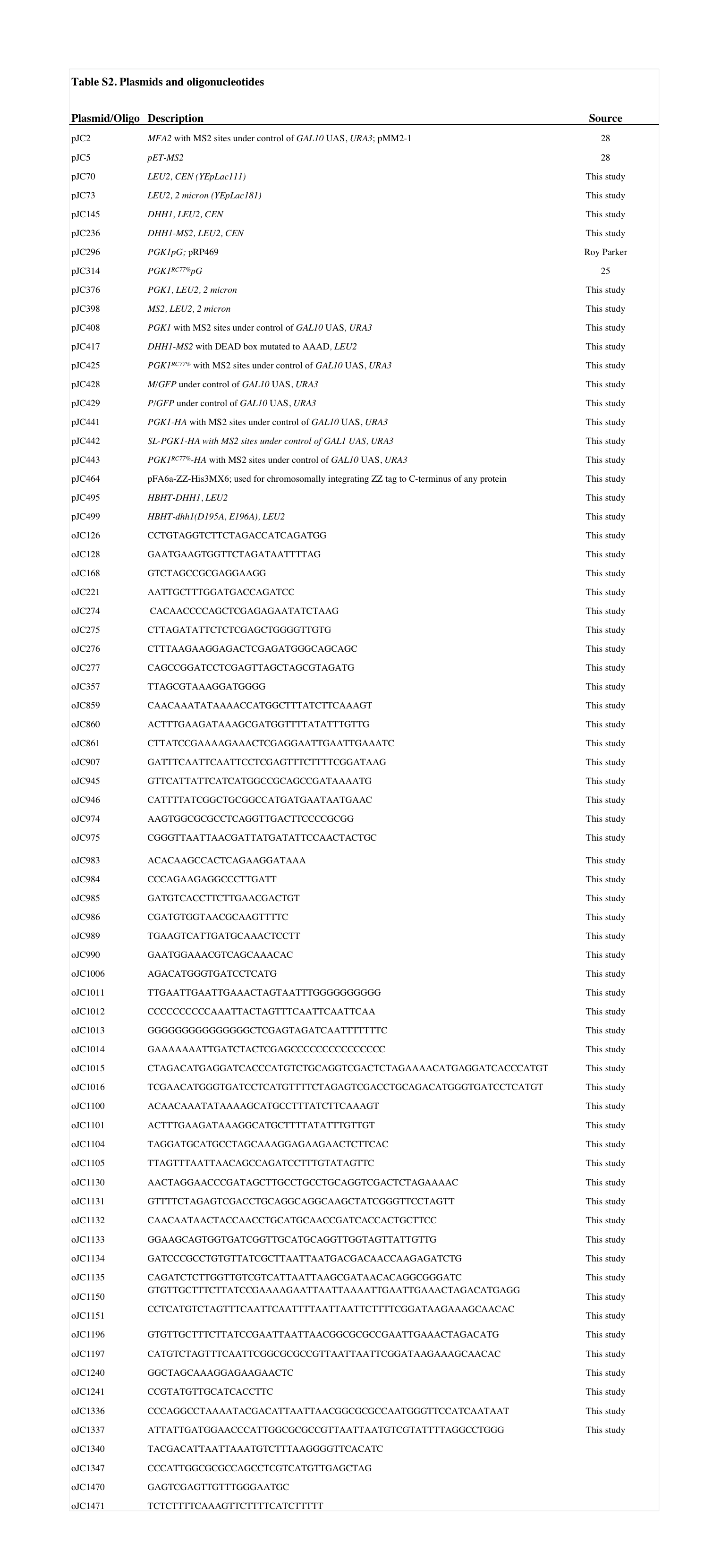

Supplement: Table S2 — Plasmids and oligonucleotides. (TIFF) [file pbio.1001342.s008.tiff]
