# Supplementary material for: The DEAD-Box Protein Dhh1 Promotes Decapping by Slowing Ribosome Movement
Source: PLoS Biol. 2012 Jun 12;10(6):e1001342. doi: 10.1371/journal.pbio.1001342 (PMC3373615; doi:10.1371/journal.pbio.1001342)
Supplement: Text S1 — Supporting methods. (DOC) [file pbio.1001342.s009.doc]

**Supporting Information for: The DEAD-box Protein Dhh1 Promotes Decapping by Slowing Ribosome Movement**

Thomas Sweet, Carrie Kovalak, and Jeff Coller*

Center for RNA Molecular Biology, Case Western Reserve University, 10900 Euclid Ave, Cleveland, OH 44106 USA

*Corresponding author: jmc71@case.edu

**Supporting Methods**

*Plasmids*

*DHH1-MS2* (pJC236) was created by cloning a PCR product containing *DHH1* (amplified with oJC126/oJC128; XbaI sites) into the XbaI site of YCpLac111. An XhoI site was added to the end of the *DHH1* ORF by site directed mutagenesis (oJC274/oJC275). *MS2* was then amplified by PCR with oJC276/oJC277 containing XhoI sites, and *MS2* was ligated into the C-terminus of *DHH1*. pJC376 was prepared by cloning *PGK1* into YEpLac181. Site directed mutagenesis was then used to add NcoI to the start codon (oJC859/oJC860) followed by XhoI to the stop codon (oJC861/oJC907). *MS2* (pJC398) was then prepared by digesting pJC376 and pET-MS2 with XhoI and NcoI; the *MS2* fragment was ligated so that it was under control of the *PGK1* promoter and terminator. *DHH1-MS2* with the DEAD box mutated to AAAD (pJC417) was created by site directed mutagenesis of pJC236 with oJC945/oJC946. *M/GFP* (pJC428) was prepared by removing an SphI site from pMM2-1 by site directed mutagenesis (oJC1130/oJC1131), adding an SphI site to the start codon (oJC1132/oJC1133), adding a PacI site (oJC1134/oJC1135), then amplifying *GFP* with oJC1104/oJC1105. *MFA2* ORF was cut out with SphI/PacI, then *GFP* ORF was ligated in. *P/GFP* (pJC429) was created by adding an SphI site to pJC408 (oJC1100/oJC1101) then a PacI site (oJC1150/oJC1151). *GFP* PCR product from above was ligated in place of the *PGK1* ORF. *PGK1-MS2* (pJC408), *SL-PGK1-MS2* (pJC424), and *PGK1^RC77%^-MS2* (pJC425) were created by creating SpeI and XhoI sites on either side of the pG tract (oJC1011/oJC1012; oJC1013/oJC1014) of pRP469 or pJC314, then ligating MS2 binding sites (oJC1015/oJC1016) in place of the pG tract. *PGK1-HA-MS2* (pJC441), *SL-PGK1-HA-MS2* (pJC442), and *PGK1^RC77%^-HA-MS2* (pJC443) were created by adding PacI and AscI sites to pJC408, pJC424, and pJC425 by site directed mutagenesis with oJC1196/oJC1197. The HA tag was then digested from *pFA6a-3HA-His3MX6* using PacI and AscI and ligated onto the C-terminus of *PGK1* ORF. *pFA6a-ZZ-His3MX6* (pJC464) was generated by digesting *pFA6a-3HA-His3MX6* with PacI and AscI, followed by ligation of PCR generated ZZ tag from the TAP tag (oJC974/oJC975). *HBHT-DHH1* (pJC495) was generated by adding PacI and AscI sites upstream of the *DHH1* start codon in pJC145 by site directed mutagenesis (pJC1336/pJC1337). PCR product of the *HBHT* tag (oJC1340/oJC1347) was then ligated in frame with *DHH1* to create an N-terminally tagged version of Dhh1.

*Assaying drug sensitivity*

Cells (yJC375 and yJC397) were grown in synthetic complete (glucose) liquid culture to saturation and then were spread evenly on synthetic complete plates to make a lawn. Once the cell suspension had dried on the plate, small discs of filter paper soaked in either dH_2_O, 50 mg/mL cycloheximide, 200 μg/mL paromomycin, or 25 mM hygromycin B were placed in the center of the plates. Cells were incubated at 24°C for several days until a distinct zone of inhibition was observed then photographed using a color digital camera.
